# The role of Si interstitials in the migration and growth of Ge nanocrystallites under thermal annealing in an oxidizing ambient

**DOI:** 10.1186/1556-276X-9-339

**Published:** 2014-07-07

**Authors:** Kuan-Hung Chen, Ching-Chi Wang, Tom George, Pei-Wen Li

**Affiliations:** 1 Department of Electrical Engineering and the Center for Nano Science and Technology, National Central University, 300 JongDa Road, ChungLi 32001, Taiwan; 2 Private Consultant, La Canada, CA 91011, USA

**Keywords:** Germanium nanocrystallites, Migration, Annealing

## Abstract

We report a unique growth and migration behavior of Ge nanocrystallites mediated by the presence of Si interstitials under thermal annealing at 900°C within an H_2_O ambient. The Ge nanocrystallites were previously generated by the selective oxidation of SiGe nanopillars and appeared to be very sensitive to the presence of Si interstitials that come either from adjacent Si_3_N_4_ layers or from within the oxidized nanopillars. A cooperative mechanism is proposed, wherein the Si interstitials aid in both the migration and coarsening of these Ge nanocrystallites through Ostwald ripening, while the Ge nanocrystallites, in turn, appear to enhance the generation of Si interstitials through catalytic decomposition of the Si-bearing layers.

## Background

Silicon-based technology is the prime enabler for high-density integrated microelectronic circuits, optoelectronics, and photovoltaic devices with ubiquitous applications ranging from mobile devices to high-end computing and communications. As Si complementary metal-oxide-semiconductor (CMOS) circuits are relentlessly scaled down to 16 nm or smaller dimensions, knowledge about fundamental nanoscopic processes in Si is becoming crucial for developing a good understanding on the limitations of nanofabrication and the development of future evolutionary directions for the technology as a whole. Many processing reactions including epitaxial growth, doping, oxidation, and silicidation are affected by the native defects in Si such as vacancies, self-interstitials, and their complexes. It is believed that Si interstitials play an important role in these processes, mostly detrimental, for instance causing such effects as undesirable transient-enhanced diffusion of dopants in *p*/*n* junctions [1,2], metal spiking at silicide/Si interfaces [3], interfacial traps along the gate oxide/Si interface [4], and stacking faults/dislocations in the epitaxial layer [1,5,6].

In this paper, we report a unique effect, hitherto unreported, that is attributable to Si interstitials present within oxide layers previously generated by the selective oxidation of polycrystalline-SiGe (poly-SiGe) nanopillars leaving behind Ge quantum dots (QDs) or nanocrystallites when the preferential oxidation of Si is complete. In this novel phenomenon, these Ge QDs or nanocrystallites appear to be very sensitive to the presence of Si interstitials, almost acting as *detectors* for these interstitial species. The mechanism appears to be complex and *long range* in comparison to the typical diffusion lengths of Si interstitials within oxide layers.

## Methods

**Figure 1:**
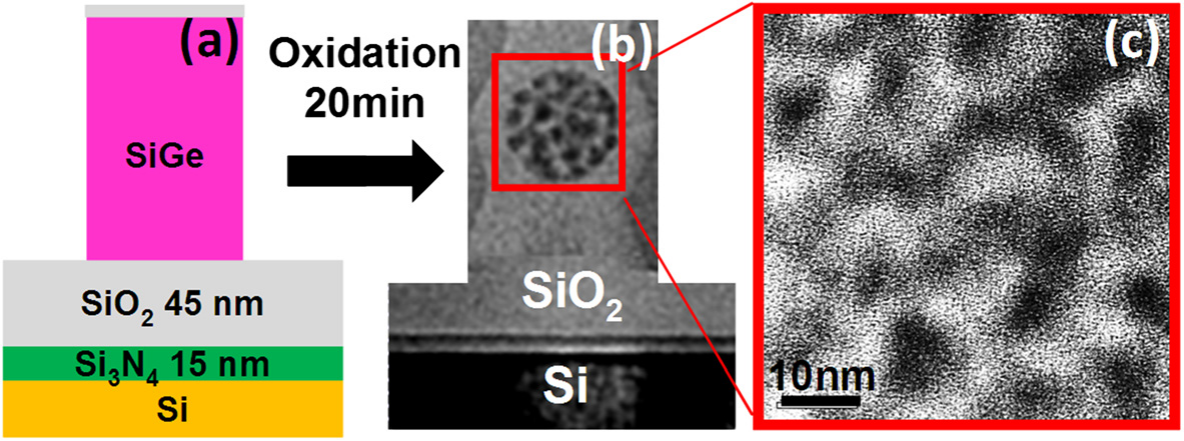
**Formation of Ge nanocrystallite clusters by thermally oxidizing poly-Si**_**0.85**_**Ge**_**0.15 **_**pillars grown over buffer oxide. (a)** Schematic diagram of the initially as-formed poly-SiGe pillars, **(b)** cross-sectional transmission electron microscopy (CTEM) micrograph of a self-assembled cluster of Ge nanocrystallites in the core of the oxidized pillars following 900°C 20 min oxidation in an H_2_O ambient, and **(c)** enlarged CTEM micrograph of the Ge nanocrystallites.

**Figure 2:**
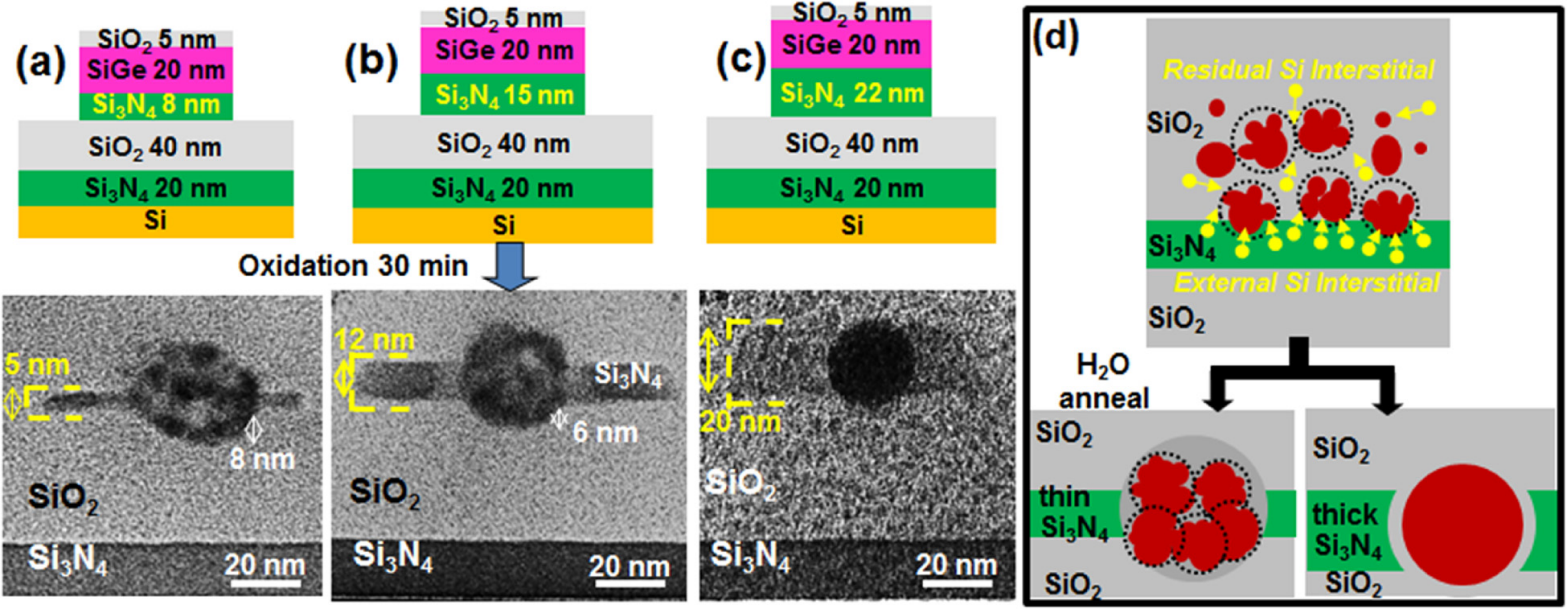
**Schematic diagrams and CTEM micrographs of Ge nanocrystallites growth and migration into underneath buffer Si**_**3**_**N**_**4**_**.** Ge nanocrystallite clusters migrate into the buffer Si_3_N_4_ underneath the original poly-Si_0.85_Ge_0.15_ pillar with coarsening and possible coalescence of these nanocrystallites after thermal annealing at 900°C for 30 min in an H_2_O ambient of the previously oxidized SiGe pillars over **(a)** 8-nm-thick, **(b)** 15-nm-thick, and **(c)** 22-nm-thick buffer Si_3_N_4_ layers. **(d)** Schematic diagram illustrating the mechanism of Si interstitials generated from the Si_3_N_4_ layers enhancing the coarsening and coalescence of Ge nanocrystallites when penetrating through thin and thick Si_3_N_4_ layers, respectively.

**Figure 3:**
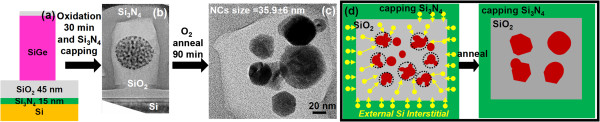
**Rapid Ge nanocrystallites coarsening in SiO**_**2 **_**without migration because of a surrounding Si**_**3**_**N**_**4 **_**capping layer.** The Si_3_N_4_ capping layer was deposited after the oxidation of the SiGe pillars to create the Ge nanocrystallite clusters and then thermally annealed at 900°C for 90 min in an O_2_ ambient. **(a)** Schematic diagram of initially as-formed poly-SiGe pillars. CTEM micrographs of **(b)** SiGe nanopillars that were thermally oxidized at 900°C for 30 min in an H_2_O ambient followed by the deposition of Si_3_N_4_ capping layer and **(c)** under further thermal annealing at 900°C for 90 min in an O_2_ ambient. **(d)** Schematic diagram showing the vertical and in-plane symmetry of Si interstitial flow that prevents the Ge nanocrystallite clusters from migrating in a preferred direction.

**Figure 4:**
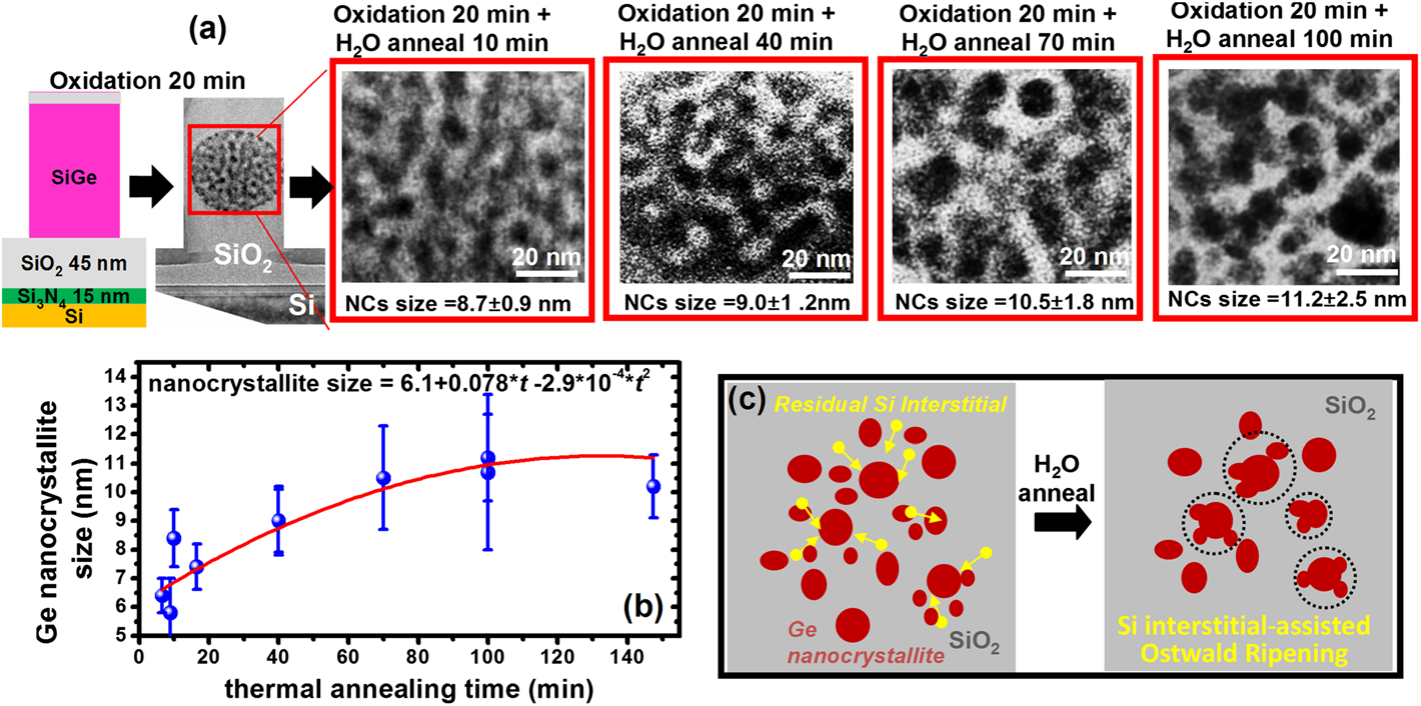
**Time evolution of Ge nanocrystallite size and coarsening under postoxidation annealing. (a)** CTEM micrographs of coarsening of the Ge nanocrystallite clusters under further thermal annealing at 900°C for various times ranging from 10 to 100 min in an H_2_O ambient. **(b)** Ge nanocrystallite size as a function thermal annealing time. The Ostwald ripening process appears to stop around an annealing time of 70 min indicative of the depletion of these residual Si interstitials. **(c)** Schematic diagram for the very slight coarsening of the Ge nanocrystallite clusters mediated by the presence of small concentrations of residual Si interstitials remaining within the oxidized poly-Si_0.85_Ge_0.15_ pillars.

Three different cases were considered for our experimental study. All cases consisted of heterostructures as shown in Figures 1,2,3,4. These samples were prepared using a CMOS-compatible approach by the deposition of poly-Si_0.85_Ge_0.15_ layers over buffer layers of Si_3_N_4_ or SiO_2_ on Si substrates using low-pressure chemical vapor deposition. In general, a multilayer deposition of Si_3_N_4_/SiO_2_/Si_0.85_Ge_0.15_/SiO_2_ was carried out sequentially on top of a Si substrate. The topmost, thin SiO_2_ layer is deposited as a hard mask for subsequent plasma etching for producing SiGe nanopillars. In one case (Figure 2), a thin layer of Si_3_N_4_ was deposited immediately prior to the deposition of the SiGe layer. The poly-Si_0.85_Ge_0.15_ layers were lithographically patterned to create nanopillar structures of various diameters (50 to 120 nm) over the buffer oxide layers and then subsequently oxidized at 900°C for 10 to 90 min to produce Ge nanocrystallites embedded within the oxide (Figure 2). It takes about 20 min to convert a 60-nm-thick, 120-nm-wide poly-Si_0.85_Ge_0.15_ pillar completely into SiO_2_/Ge nanocrystallites at 900°C by thermal oxidation within an H_2_O ambient. The entire process has been described together with the mechanism for Ge nanocrystallite formation in previous publications [7-9]. For yet another sample (Figure 3), the oxidized pillars were subsequently encapsulated via the conformal deposition of a thin *capping layer* of Si_3_N_4_. Details of the thicknesses of the various layers are provided in the schematic diagrams of various structures. It is our contention that Si interstitials are provided both by the Si_3_N_4_ layers and by the oxidized SiGe nanopillars themselves, in the latter case, perhaps generated by the incomplete oxidation of the Si within the SiGe.

## Results and discussion

The experimental procedure for the formation of Ge nanocrystallite cluster within SiO_2_ is described schematically in Figure 1. The SiO_2_ capping layer prevents the evaporation of Ge during the final, high-temperature oxidation process for the generation of Ge QDs from the SiGe layer. The bottom Si_3_N_4_ layer (in contact with the Si substrate) also acts as an oxidation mask to protect the Si substrate from oxidation during the thermal oxidation of the SiGe nanopillars. Thermal oxidation preferentially converts the Si from the poly-Si_0.85_Ge_0.15_ into SiO_2_, while squeezing the Ge released from solid solution within each poly-SiGe grain into irregularly shaped Ge nanocrystallite clusters that ostensibly assume the crystal orientation and the morphology of the original poly-SiGe grains. Thus, within this newly formed SiO_2_, a self-assembled cluster of Ge nanocrystallites appears in the core of the oxidized nanopillars (Figure 1) and the Ge nanocrystallites are 5.8 ± 1.2 nm in size with an interspacing of approximately 4.8 nm [7].

The first evidence of a unique *growth and migration* behavior mediated by the presence of Si interstitials was observed in the sample that contained a thin Si_3_N_4_ layer directly below the original SiGe nanopillar (Figure 2) and which was subjected, following oxidation of the poly-Si_0.85_Ge_0.15_ layer, to further thermal annealing at 900°C for 30 min in an H_2_O ambient. The entire cluster of Ge nanocrystallites appears to migrate from its original location within the oxide and ultimately *penetrates* the Si_3_N_4_ layer. We believe that this is because of the Si_3_N_4_ layer acting as an initial, local source of Si interstitials via a catalytic decomposition process described elsewhere [9,10]. In brief, the Ge nanocrystallite clusters/QDs migrate through the underlying Si_3_N_4_ layer in a two-step catalytic process, during which the QDs first enhance the local decomposition of the Si_3_N_4_ layer, releasing Si that subsequently migrates to the QDs. In the second step, the Si rapidly diffuses and is ultimately oxidized at the distal surface of the QDs, generating the SiO_2_ layer behind the QDs and thus facilitating the deeper penetration of the QDs in the Si_3_N_4_ layer. It is clearly seen in Figure 2 that an increase in the layer thickness of Si_3_N_4_ in proximity to the SiGe nanopillars enhances the initial, local source of Si for facilitating the migration of the as-formed Ge nuclei after the SiGe pillar is oxidized. The increased Si content results in a considerable enhancement in the coarsening of the Ge nanocrystallites, as observed when increasing the thickness of buffer Si_3_N_4_ from 8 to 15 nm (Figure 2a,b), and also serves to achieve complete coalescence of the nanocrystallites to form a single Ge QD when the buffer Si_3_N_4_ is thick enough (22 nm) (Figure 2c). Attendant to the migration process are changes that occur to the crystallographic morphology, crystallinity, and sizes of the Ge nanocrystallites. Thus, the Ge nanocrystallites are undergoing an Ostwald ripening process [11] which also, in addition to the migration, appears to be facilitated by the Si interstitials.

Further evidence of the Si interstitial-mediated Ostwald ripening process was provided by the sample with the Si_3_N_4_ capping layer (Figure 3) subjected to thermal annealing at 900°C for 90 min in an H_2_O ambient. In this case, the Ge nanocrystallite clusters within the pillars experience lateral Si interstitial fluxes in all azimuthal directions because of the surrounding Si_3_N_4_. Therefore, the in-plane symmetry of the radial Si interstitial fluxes prevents the Ge nanocrystallite clusters from adopting any one, particular direction for preferential migration as was seen in the previous case (Figure 2). However, the Ostwald ripening proceeds unhindered and results in significant coarsening of the Ge nanocrystallites by as much as 3 to 4 × !

With the profound understanding gained by the above two cases, we can now examine the case of the nanopillar sample itself, without either the underlying Si_3_N_4_ layer or the Si_3_N_4_ capping layer but also subjected to the same thermal annealing at 900°C for various times within an H_2_O ambient. In this case, it is observed that the Ostwald ripening process occurs at a much slower rate with a slight change in the average size of the Ge nanocrystallites within the cluster. Starting from an original average size of 5.8 ± 1.2 nm for the as-formed Ge nanocrystallites, Figure 4a shows the time evolution of the Ge nanocrystallite clusters formed after thermal annealing at 900°C under an H_2_O ambient of 120-nm-diameter pillars of previously oxidized Si_0.85_Ge_0.15_ for annealing times of 10, 40, 70, and 100 min, respectively. The average nanocrystallite size changes from approximately 7 nm at 10 min of annealing to 8.7 ± 0.9 nm at 40 min, 10.5 ± 1.8 nm at 70 min, and 11.2 ± 2.5 nm at 100 min of annealing (Figure 4b). Based on the above evidence, we believe that the slight coarsening of the Ge nanocrystallites that is observed with increased annealing times is mediated by the small, residual concentration of Si interstitials left behind after thermal oxidation of the SiGe layer. The Ostwald ripening process essentially stops around 70 min when these interstitials are used up, i.e., converted to oxide.

The above TEM observations clearly reveal that the growth and migration behaviors of Ge nanocrystallites are very sensitive to the presence and the content of Si interstitials that are provided either externally by adjacent Si_3_N_4_ layers or by small concentrations of residual Si interstitials remaining within the oxidized poly-SiGe pillars. The role of Si interstitials in the growth of Ge nanocrystallites under thermal annealing in an oxidizing ambient is sketched in Figures 2d, 3d, and 4c. Although a large body of work exists in the literature on the generation and role of Si interstitials, to our knowledge, the above phenomenon has never been reported before. Previous work has attributed the thermal oxidation of Si inducing a drastic lateral expansion of the silicon lattice [12] and the generation of silicon self-interstitials as a means of partially relieving the compressive stress in the growing oxide layer that develops as a result of a 2.25× volume expansion when Si is converted to SiO_2_. The majority of these Si interstitials generated during Si oxidation diffuse into the growing oxide layer and are also oxidized [13,14], while a relatively small, but significant, amount of interstitials diffuse into the Si substrate, causing supersaturation of these interstitials and the consequent precipitation as oxidation stacking faults (OSFs) [5,6] or oxidation-enhanced diffusion (OED) [1,2] of some dopants. Interestingly, the OED of boron during the thermal oxidation of Si is effectively suppressed through the introduction of a thin layer of Si_1 - *x*_Ge_*x*_ or Si_1 - *x*_Ge_*x*_C_*y*_ over the Si substrate or even completely eliminated when the Ge or C concentration is high [15-17]. Moreover, the reduction of the Si interstitials has been shown to be Ge concentration dependent. Again, to our knowledge, we have not found previous work describing a *cooperative* mechanism, wherein the Si interstitials aid in both the migration of Ge nanocrystallites and in the coarsening of these nanocrystallites through Ostwald ripening as clearly shown above. The additional, interesting aspect of this novel mechanism is that as described by us previously [9,10], the Ge nanocrystallites also appear to enhance the decomposition of the Si-bearing Si_3_N_4_ layers resulting in further generation of Si interstitials.

The quality of the oxide generated by the thermal oxidation of the poly-Si_0.85_Ge_0.15_ could also play a significant role in facilitating the new mechanism that we have discovered. Diffusion lengths of Si interstitials in SiO_2_ calculated at 900°C for diffusion times of 10, 40, 70, 100, and 145 min are 0.72, 1.43, 1,89, 2.26, and 2.72 nm, respectively, based on the equation of *D* = 1.2 × 10^-9^⋅exp(-1.9/*k*_*B*_*T*) [18]. Obviously, these diffusion lengths are too small to explain the Si interstitial-mediated mechanism that we have observed. Hence, we believe that the oxide generated from poly-Si_0.85_Ge_0.15_ is possibly not as dense as the conventional, thermally generated oxide from Si substrates and therefore allows the faster diffusion of the Si interstitials through the oxide.

## Conclusions

In conclusion, we have observed a unique phenomenon of the migration and growth of Ge nanocrystallite clusters within SiO_2_ layers that is made possible by the presence of Si interstitials during high-temperature thermal annealing in an oxidizing ambient. The Ge nanocrystallites generated by selective oxidation of SiGe appear to be very sensitive to the presence of Si interstitials that are provided either by adjacent Si_3_N_4_ layers or by residual Si interstitials left behind after thermal oxidation of the SiGe. The Si interstitials also facilitate the Ostwald ripening of the Ge nanocrystallites. We have proposed a novel cooperative mechanism for this Si interstitial-mediated growth and migration of Ge nanocrystallites under thermal oxidation. We envisage further scientific exploration of this unique phenomenon and the demonstration of new device geometries with Ge QDs buried within various Si-containing layers.

## Competing interests

The authors declare that they have no competing interests.

## Authors’ contributions

KHC and CCW carried out the Ge QD growth and TEM experimentation and analysis. TG conceived the mechanism of Ge QD migration and drafted the manuscript. PWL conceived the study, supervised the work, and contributed to the data analysis and manuscript preparation. All authors read and approved the final manuscript.
